# Identification and serological characterization of MtbAg1073, a conserved *Mycobacterium tuberculosis* complex antigen recognized in active tuberculosis

**DOI:** 10.1265/ehpm.26-00071

**Published:** 2026-07-10

**Authors:** Khouanheuane Sengkhamyong, Pheophet Lamaningao, Takaki Shimono, Sengthong Seumlamvanh, Seiji Kanda

**Affiliations:** 1 Department of Hygiene and Public Health, Kansai Medical University, 2-5-1 Shinmachi, Hirakata, Osaka 573-1010, Japan; 2 Khammouane Provincial Health Department, Souphanouvong Ave, Ban Laophosay, Thakhek District, Khammouane Province, Lao PDR

**Keywords:** Tuberculosis, *Mycobacterium tuberculosis* complex, Immunoscreening, Serological diagnosis, Antigen discovery, Dot-blot assay, MtbAg1073

## Abstract

**Background:**

Tuberculosis (TB) remains a major global health challenge, with delayed diagnosis perpetuating transmission in resource-limited settings. Although the sensitivity of molecular diagnostic methods is high, their dependence on the laboratory infrastructure limits accessibility in many regions. Serological approaches that detect host antibody responses represent a technically simple diagnostic strategy; however, the performance of currently available TB serodiagnostic tests is inconsistent, largely due to suboptimal antigen selection and heterogeneity in host immune responses. Therefore, the identification of conserved antigens naturally recognized during human infection is essential for improving serological diagnostics.

**Methods:**

A cDNA expression library derived from *Mycobacterium bovis* BCG Tokyo was constructed and screened by colony immunoblotting using pooled sera from patients with active TB. Immunoreactive clones were sequenced and analyzed bioinformatically. The full-length coding sequence of the identified antigen was expressed recombinantly using a cold-shock expression system in *Escherichia coli*. The recombinant protein was purified and evaluated for immunoreactivity using a Western blot analysis. Serological reactivity was further assessed using a dot-blot assay with individual serum samples from microbiologically confirmed TB patients (n = 40) and healthy controls (n = 40) from the Lao People’s Democratic Republic. Diagnostic performance was evaluated by a receiver operating characteristic (ROC) curve analysis.

**Results:**

Immunoscreening identified a conserved hypothetical protein corresponding to Rv1073 of *M. tuberculosis*, designated MtbAg1073, for which no antigenicity had been previously reported. The protein was highly conserved among members of the *M. tuberculosis* complex. Recombinant expression using a cold-shock system produced soluble His-tagged MtbAg1073, which was specifically recognized by sera from TB patients in the Western blot analysis. Dot-blot assays showed significantly higher antibody responses in TB patients than in controls (median intensity: 761.2 vs. 471.9; the Mann–Whitney U test, *P* = 0.0002). The ROC analysis demonstrated moderate discriminatory performance (AUC = 0.734; 95% CI: 0.624–0.844), with a sensitivity of 70.0% and specificity of 67.5% at the optimal cut-off value.

**Conclusions:**

MtbAg1073 is a conserved and immunoreactive antigen that elicits detectable antibody responses in active TB. These findings provide preliminary evidence of its immunoreactivity and warrant further investigation into its potential utility in serological approaches.

**Supplementary information:**

The online version contains supplementary material available at https://doi.org/10.1265/ehpm.26-00071.

## Graphical Abstract

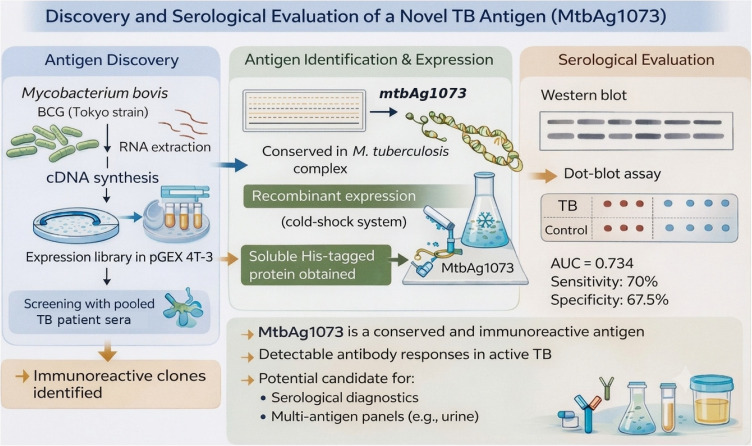

## Background

Tuberculosis (TB) remains one of the leading causes of death from a single infectious agent worldwide, with an estimated 10.7 million new cases and 1.23 million deaths being reported in 2022 [1, 2]. Despite substantial advances in molecular diagnostic technologies, delayed case detection continues to sustain transmission, particularly in low- and middle-income countries [3]. Although the sensitivities of nucleic acid amplification tests and culture-based methods are high, their reliance on the laboratory infrastructure, technical expertise, and sample quality limits universal accessibility. Therefore, there remains a strong need for complementary diagnostic approaches that are biologically informative, technically simple, and adaptable to diverse clinical settings [4].

Serological approaches that detect host antibody responses represent an attractive diagnostic strategy because of their simplicity and scalability. Various *Mycobacterium tuberculosis*–derived antigens, including secreted proteins, such as MPB64, and cell wall components, such as lipoarabinomannan (LAM), have been examined as targets for an immunological diagnosis. Although these antigen-based assays have shown diagnostic utility in certain clinical contexts, their performance has been inconsistent across populations, stages of disease, and geographical settings [5, 6]. Furthermore, the sensitivity and specificity of many previously developed TB serological tests have been inconsistent, leading the World Health Organization to discourage the use of currently available commercial antibody-based TB serodiagnostic tests [7].

The limited performance of these assays has been largely attributed to two interrelated factors: suboptimal antigen selection and heterogeneity in host immune responses [5]. Many previously evaluated antigens were not selected based on the systematic validation of immunoreactivity in human disease, but were instead identified through observations of immunogenicity in culture filtrate proteins or animal models. In addition, strain-specific polymorphisms have been reported for certain antigens, and variations among circulating *M. tuberculosis* lineages may affect host immune responses [5]. These factors may contribute to variability in the performance of serological diagnostics across different populations.

To address these challenges, approaches that identify antigens recognized by the human humoral immune response during active TB are required. Immunoscreening of mycobacterial expression libraries using patient-derived sera represents an effective strategy for discovering these antigens. Unlike candidate-based approaches that rely on previous assumptions regarding antigen immunogenicity, this method enables the discovery of previously uncharacterized proteins associated with active disease. In addition, antigens identified through immunoscreening must be evaluated not only for immunoreactivity, but also for the feasibility of recombinant expression and purification, as soluble protein production is a prerequisite for reliable immunoassay development and insoluble aggregates may compromise epitope integrity and assay performance [8, 9].

At the early stage of an antigen evaluation, dot-blot analysis represents a practical method for the semi-quantitative comparisons of antibody responses using small amounts of antigens and requiring limited assay optimization [10–12].

However, few studies have systematically integrated antigen discovery with the evaluation of recombinant expression feasibility and preliminary serological evaluation using clinical samples.

In the present study, we constructed a complementary DNA (cDNA) expression library derived from *M. bovis* Bacillus Calmette–Guérin (BCG) Tokyo and performed immunoscreening using sera from patients with active TB. Through this approach, we identified a previously uncharacterized and highly conserved antigen within the *M. tuberculosis* complex (MTBC), which we provisionally designated MtbAg1073. We further evaluated its sequence conservation, recombinant expression characteristics, and serological reactivity using Western blot and dot-blot analyses of a cohort from the Lao People’s Democratic Republic (PDR). By integrating antigen discovery, expression feasibility, and an early-stage serological evaluation, this study aimed to identify diagnostically relevant antigens.

## Methods

### 1 Serum samples

Regarding immunoscreening of the cDNA library, pooled serum samples were obtained from Japanese patients with microbiologically confirmed active TB. Pooled sera from healthy volunteers without a history of TB were used as controls.

In the antigenicity evaluation, a total of 87 serum samples (45 TB patients and 42 non-TB controls) were included in this study for dot-blot analysis from a larger cohort of serum samples collected in Lao PDR. TB patient sera were obtained at Khammouane Provincial Hospital, a high TB-burden area. Patients diagnosed with active pulmonary TB were included in this study, and the diagnosis was microbiologically confirmed using the GeneXpert MTB/RIF assay in accordance with national diagnostic guidelines. After data quality control and outlier exclusion, 80 samples (40 TB patients and 40 controls) were included in the final analysis.

Control sera were collected from asymptomatic village residents in Khammouane Province who had no clinical symptoms suggestive of TB and no known history of a TB diagnosis or treatment. No additional microbiological or immunological testing for TB, including interferon-γ release assays, a sputum examination, or molecular tests, was performed for control participants.

All serum samples were aliquoted immediately after collection, stored at −30 °C, and transported under cold chain conditions to Kansai Medical University (Osaka, Japan) for analysis. Pooled and individual serum samples were both used in Western blot and dot-blot analyses.

### 2 Bacterial strain

The *M. bovis* BCG Tokyo strain and genomic DNA used in this study were kindly supplied by Dr. Takemitsu Matsuo.

### 3 Construction of the cDNA expression library

Total RNA was extracted from *M. bovis* BCG Tokyo cultured to the logarithmic growth phase using TRIzol reagent (Invitrogen; Thermo Fisher Scientific, Inc., Waltham, MA, USA) according to the manufacturer’s instructions. cDNA was synthesized from total RNA by reverse transcription using ReverTra Ace (Toyobo Co., Ltd., Osaka, Japan) with random primers. Double-stranded cDNA was generated and ligated to a pre-annealed EcoRI–NotI–BamHI adaptor (Takara Bio Inc., Shiga, Japan, Code No. 4510). Adaptor-ligated fragments were digested with EcoRI and NotI and cloned into the corresponding restriction sites of the pGEX-4T-3 expression vector (Cytiva, Marlborough, MA, USA) to enable in-frame fusion with glutathione S-transferase (GST). Recombinant plasmids were transformed into *E. coli* competent cells to establish a cDNA expression library.

### 4 Immunoscreening of the cDNA library

The cDNA expression library was screened by colony immunoblotting. Briefly, approximately 6,000 independent transformed *E. coli* colonies were transferred onto nitrocellulose membranes and incubated until visible colony formation. The expression of GST-fusion proteins was induced with isopropyl β-D-1-thiogalactopyranoside (IPTG) at a final concentration of 0.1 mM at 37 °C for 3–5 hours.

Membranes were blocked with 5% skim milk in Tris-buffered saline containing 0.1% Tween-20 (TBST) at room temperature for 1 hour and then incubated with pooled serum from Japanese patients with active TB (diluted 1:100 in blocking buffer) as the primary antibody at room temperature for 2 hours. After washing three times with TBST, bound antibodies were detected using a horseradish peroxidase (HRP)-conjugated anti-human IgG secondary antibody (catalog no. 5220-0330; SeraCare Life Sciences Co., Milford, MA, USA; diluted 1:10,000 in blocking buffer). Signals were visualized using 3,3′-diaminobenzidine (DAB) substrate (FUJIFILM Wako Pure Chemical Corp., Osaka, Japan).

Positive clones were isolated, replated at a lower density, and subjected to secondary screening to confirm the reproducibility of immunoreactivity.

### 5 Sequence analysis and bioinformatics

Plasmid DNA was extracted from positive clones using standard alkaline lysis procedures. Insert sequences were elucidated by Sanger sequencing using vector-specific primers on an ABI automated DNA sequencer (Applied Biosystems, Thermo Fisher Scientific).

Nucleotide sequence similarity searches were performed using BLASTn, and amino acid sequence similarity was assessed using BLASTp programs available through the National Center for Biotechnology Information (NCBI). Based on the results obtained, gene-specific primers were designed to amplify the full-length coding sequence of the identified gene. PCR amplification was performed using cDNA derived from *M. bovis* BCG Tokyo as the template, and the amplified product was confirmed by sequencing.

A conserved domain analysis was performed using the NCBI Conserved Domain Database (CDD) [13]. Signal peptide prediction and transmembrane topology were assessed using SignalP (version 5.0) [14] and TMHMM (version 2.0) [15], respectively.

### 6 Recombinant protein expression and purification

The full-length coding sequence was amplified and subcloned into the pCold ProS2 expression vector (Takara Bio Inc., Shiga, Japan) to generate an N-terminal His-ProS2 fusion construct. The recombinant plasmid was transformed into *E. coli* BL21 (DE3) cells.

Protein expression was induced with IPTG at a final concentration of 0.1 mM at 15 °C for 24 hours according to the manufacturer’s instructions. After induction, bacterial cells were harvested and lysed by sonication in EzBactYeast lysis buffer (ATTO Co., Ltd., Tokyo, Japan).

The soluble fraction was obtained by centrifugation at 15,000 × *g* for 20 minutes and applied to a Ni-Sepharose affinity resin (Cytiva, Marlborough, MA, USA). After washing, the His-tagged recombinant protein was eluted with 500 mM imidazole-containing buffer. Purified protein fractions were analyzed by sodium dodecyl sulfate–polyacrylamide gel electrophoresis (SDS-PAGE) to confirm purity and the expected molecular weight, and were then stored at −30 °C until used.

### 7 Western blot analysis

The purified recombinant His-tagged protein was separated by SDS-PAGE using an 8% polyacrylamide gel (approximately 5 µg per lane) and subsequently transferred onto nitrocellulose membranes (GVS North America Inc., Sanford, ME, USA) using a semi-dry blotting apparatus (Horizon Blot AE-6677, ATTO Co., LTD.). Protein transfer was performed in transfer buffer containing 48 mM Tris, 39 mM glycine, and 10% methanol at a constant current of 90 mA for 30 minutes.

Membranes were blocked with Ez Block reagent (ATTO Co., LTD.) at room temperature for 1 hour. To confirm recombinant protein expression, membranes were incubated with an anti-His-tag monoclonal antibody (clone AD1.1.10; Santa Cruz Biotechnology Inc., Dallas, TX, USA; 1:1,000 in blocking buffer). To evaluate immunoreactivity, membranes were incubated with pooled or individual serum samples from TB patients or control subjects (diluted 1:100 in blocking buffer). All primary antibody incubations were performed at room temperature for 2 hours followed by an overnight incubation at 4 °C.

After three washes with TBS-T (0.1% Tween-20), membranes were incubated with HRP-conjugated secondary antibodies at room temperature for 2 hours: anti-mouse IgG-HRP (catalog no. 5220-0341; SeraCare Life Sciences; 1:10,000) for His-tag detection or anti-human IgG-HRP (catalog no. P021402-2; Dako, Glostrup, Denmark; 1:15,000) for serum reactivity. Immunoreactive signals were visualized using tetramethylbenzidine substrate (catalog no. 18186-24; Nacalai Tesque, Inc., Kyoto, Japan) according to the manufacturer’s instructions.

### 8 Dot-blot analysis

The recombinant His-tagged protein (100 ng per well in 200 µL) was spotted in duplicate onto nitrocellulose membranes using a Bio-Dot 96-well microfiltration apparatus (Bio-Rad Laboratories, Inc., Hercules, CA, USA). After sample application, membranes were blocked with Ez Block reagent (ATTO Co., LTD.) at room temperature for 1 hour.

Membranes were incubated with pooled or individual serum samples from TB patients or control subjects (diluted 1:1,000 in blocking buffer) at room temperature for 2 hours followed by an overnight incubation at 4 °C. After three washes with TBS-T, membranes were incubated with the HRP-conjugated rabbit anti-human IgG antibody (1:10,000 in blocking buffer) at room temperature for 2 hours.

Immunoreactive signals were visualized using an enhanced chemiluminescence substrate (Bio-Rad Laboratories, Inc.) and detected with a Fusion S Solo imaging system (Vilber, Collégien, France). Signal intensities were quantified using ImageJ software (version 1.54g, National Institutes of Health, Bethesda, MD, USA) with the Band/Peak Quantification Tool macro [16]. Circular regions of interest of identical sizes were placed over each dot, and integrated density values were measured. Background-corrected signal intensities were used for a comparative analysis. Two technical replicates were analyzed for each serum sample and the mean value was used in subsequent statistical analyses.

Because not all samples could be processed simultaneously, dot-blot assays were performed in multiple independent runs (four runs in total). To control for inter-assay variability, pooled TB serum samples were included in each run as an internal control. Signal intensities were normalized across runs by applying a scaling factor calculated as 1000 divided by the mean signal intensity of the pooled TB control (average of duplicate measurements), which was then multiplied by the signal intensity of each individual sample. Quantitative analysis of dot-blot signal intensities was performed using individual serum samples.

### 9 Statistical analysis

Statistical analyses were performed using GraphPad Prism version 9.0 (GraphPad Software, San Diego, CA, USA). Comparisons between the TB and control groups were performed using the non-parametric Mann–Whitney U test (two-tailed).

A receiver operating characteristic (ROC) curve analysis was performed to evaluate diagnostic performance. The primary cut-off value was selected using the point closest to the upper-left corner of the ROC curve. Sensitivity, specificity, and the area under the ROC curve (AUC) were calculated with 95% confidence intervals (CI). A p-value <0.05 was considered to be significant. Age and sex distributions between groups were compared using the Mann–Whitney U test and Fisher’s exact test, respectively.

Samples exhibiting technical artifacts that impeded reliable signal quantification—such as excessively high background signals, overlapping noise signals, or uneven signal distribution—were excluded prior to quantitative analysis. For samples with a coefficient of variation (CV) greater than 15% between duplicate measurements, signal quality was assessed by visual inspection, and samples were excluded only when reliable quantification was not possible. Subsequently, outlier detection was conducted using the Grubbs’ test as implemented in GraphPad Prism version 9.0, and identified outliers were excluded from the final analysis (two TB samples and one HC sample).

The sample selection process, including exclusion criteria and the number of excluded samples, is summarized in Fig. S1.

## Results

### 1 Identification of immunoreactive clones

The demographic characteristics of the study participants are summarized in Table 1. The TB group was significantly older and had a higher percentage of male participants than the control group.

**Table 1:** Demographic characteristics of study participants.

**Characteristic**	**HC (n = 40)**	**TB (n = 40)**	**p-value**
Age, years (mean ± SD)	43.3 ± 22.8	54.9 ± 17.1	0.013
Male, n (%)	16 (40.0)	26 (65.0)	0.043

Age distributions were compared using the Mann–Whitney U test. Sex distributions were compared using Fisher’s exact test. HC: healthy controls; TB: tuberculosis patients.

The *M. bovis* BCG Tokyo cDNA expression library was screened by colony immunoblotting using pooled serum from patients with active TB. Multiple DAB-positive colonies were identified and isolated for further analysis. Secondary screening confirmed the reproducibility of positive clones.

Plasmid DNA was extracted from candidate clones, and the presence of inserts was confirmed by PCR, revealing that 15 clones contained cDNA inserts. The sequence analysis showed that all inserts were distinct; however, several corresponded to non-coding or truncated regions. One clone consistently exhibited strong and reproducible immunoreactivity and was selected for subsequent characterization.

### 2 Identification of the full-length open reading frame (ORF)

A sequence analysis of the selected immunoreactive clone initially yielded a 288-bp partial insert. A BLASTn analysis against the NCBI database identified a corresponding full-length ORF of 852 bp, including the confirmed start and stop codons, located at positions 1199867–1200718 in the genome of *M. tuberculosis* variant bovis BCG strain Tokyo 172 (GenBank accession CP014566.1; locus tag AZH48_05705; Protein ID AMO12297.1).

Based on this information, gene-specific primers were designed to amplify the complete coding sequence from *M. bovis* BCG Tokyo cDNA. PCR amplification successfully generated an 852-bp fragment, which was subsequently confirmed by Sanger sequencing. The amplified sequence showed 100% nucleotide identity (852/852 bp) to the corresponding locus in BCG Tokyo 172.

The ORF encodes a 283-amino-acid protein with a calculated molecular mass of approximately 31 kDa. The deduced amino acid sequence showed broad conservation across representative members of the MTBC, including *M. tuberculosis* H37Rv (AL123456.3), *M. tuberculosis* variant bovis BCG str. Tokyo 172 (CP014566.1), *M. bovis* AF2122/97 (CP133604.1), and *M. africanum* UT307 (CP014617.1), as demonstrated by the BLASTp analysis.

This gene was provisionally designated *mtbAg1073* in the present study, reflecting its correspondence to Rv1073 in the *M. tuberculosis* H37Rv reference strain, and the encoded protein was hereafter referred to as MtbAg1073.

### 3 Bioinformatic characterization of MtbAg1073

To further characterize MtbAg1073, *in silico* analyses were performed. A BLASTp analysis of the deduced 283-amino-acid sequence confirmed broad conservation across representative members of the MTBC.

A conserved domain analysis using NCBI CDD identified a C-terminal region (amino acids 174–275) showing significant similarity to the YcjD family (COG2852), annotated as a very-short-patch-repair endonuclease–like protein (E-value = 3.76 × 10^−13^). Weaker similarity was observed in the N-terminal region (amino acids 44–173) to the AbiEi family (COG5340), a component of abortive phage infection systems (E-value = 5.87 × 10^−4^). Despite these similarities, MtbAg1073 remains annotated as a conserved hypothetical protein in current genomic databases.

The prediction of subcellular features using SignalP 5.0 revealed no detectable signal peptide, and an analysis with Phobius identified no transmembrane helices. These results suggest that MtbAg1073 is unlikely to be a classical secreted or membrane-associated protein.

### 4 Recombinant expression, purification, and immunoreactivity of MtbAg1073

Initial attempts to express recombinant MtbAg1073 using a GST-fusion expression system (pGEX-4T-3 vector) resulted predominantly in insoluble protein aggregates. To improve protein solubility, the *mtbAg1073* gene was subsequently subcloned into the pCold ProS2 vector and expressed in *E. coli* using a cold-shock expression system.

**Fig. 1:**
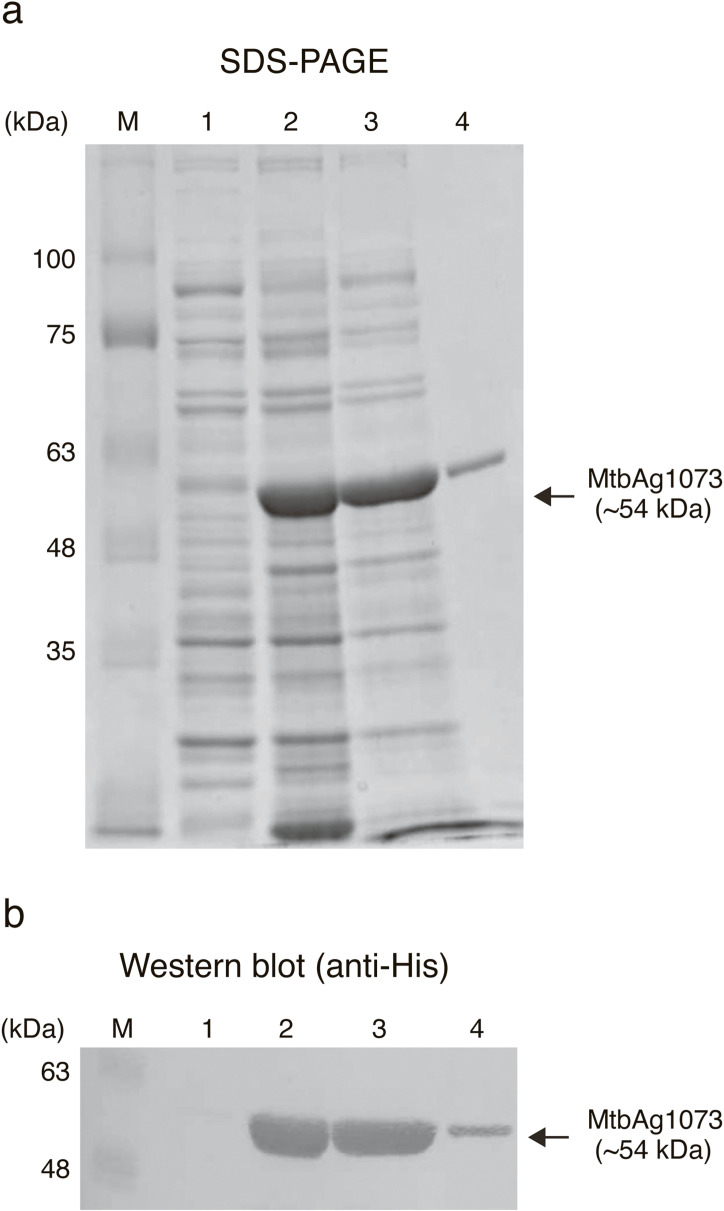
Expression of recombinant MtbAg1073 in *Escherichia coli*. (a) SDS-PAGE analysis of recombinant MtbAg1073 expression. (b) Western blot analysis using an anti-His tag antibody confirming the expression of the recombinant protein. M: molecular weight marker; lane 1: cells before IPTG induction; lane 2: whole-cell lysate after IPTG induction; lane 3: soluble protein fraction after sonication; lane 4: insoluble pellet fraction after sonication. The expected band corresponding to MtbAg1073 (∼54 kDa) is indicated by an arrow.

Expression was induced at 15 °C, and under these low-temperature conditions, the majority of the His-ProS2-tagged recombinant protein was recovered in the soluble fraction (Fig. 1).

**Fig. 2:**
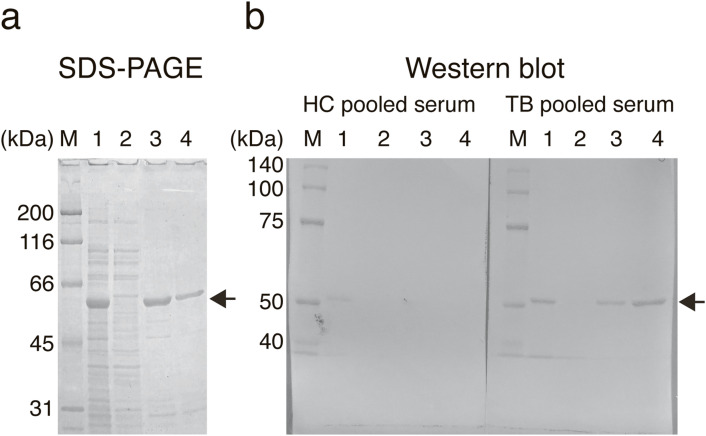
Purification and immunoreactivity of recombinant MtbAg1073. (a) SDS-PAGE analysis of purified recombinant MtbAg1073. (b) Western blot analysis using pooled healthy control and pooled tuberculosis patient sera. M: molecular weight marker; lane 1: soluble fraction; lane 2: flow-through fraction; lane 3: first elution fraction; lane 4: second elution fraction. A specific band corresponding to MtbAg1073 (∼54 kDa) was detected in pooled TB patient sera, but not in pooled healthy control sera.

The recombinant protein was purified from the soluble lysate using Ni-Sepharose affinity chromatography. A SDS-PAGE analysis revealed a predominant band at the expected molecular mass of approximately 54 kDa (Fig. 2A), corresponding to the MtbAg1073 protein (∼31 kDa) fused to the His-ProS2 tag (∼23 kDa). The purity of the recombinant protein was estimated to exceed 90% based on a visual inspection of the SDS-PAGE gel.

A Western blot analysis confirmed the identity of the recombinant protein using an anti-His tag antibody, which detected a single band at the expected molecular weight (Fig. 1B). When probed with pooled sera from TB patients, a clear immunoreactive band was observed at approximately 54 kDa, whereas no detectable signal was observed with pooled healthy control (HC) sera (Fig. 2B).

### 5 Optimization of antigen loading for a dot-blot analysis

**Fig. 3:**
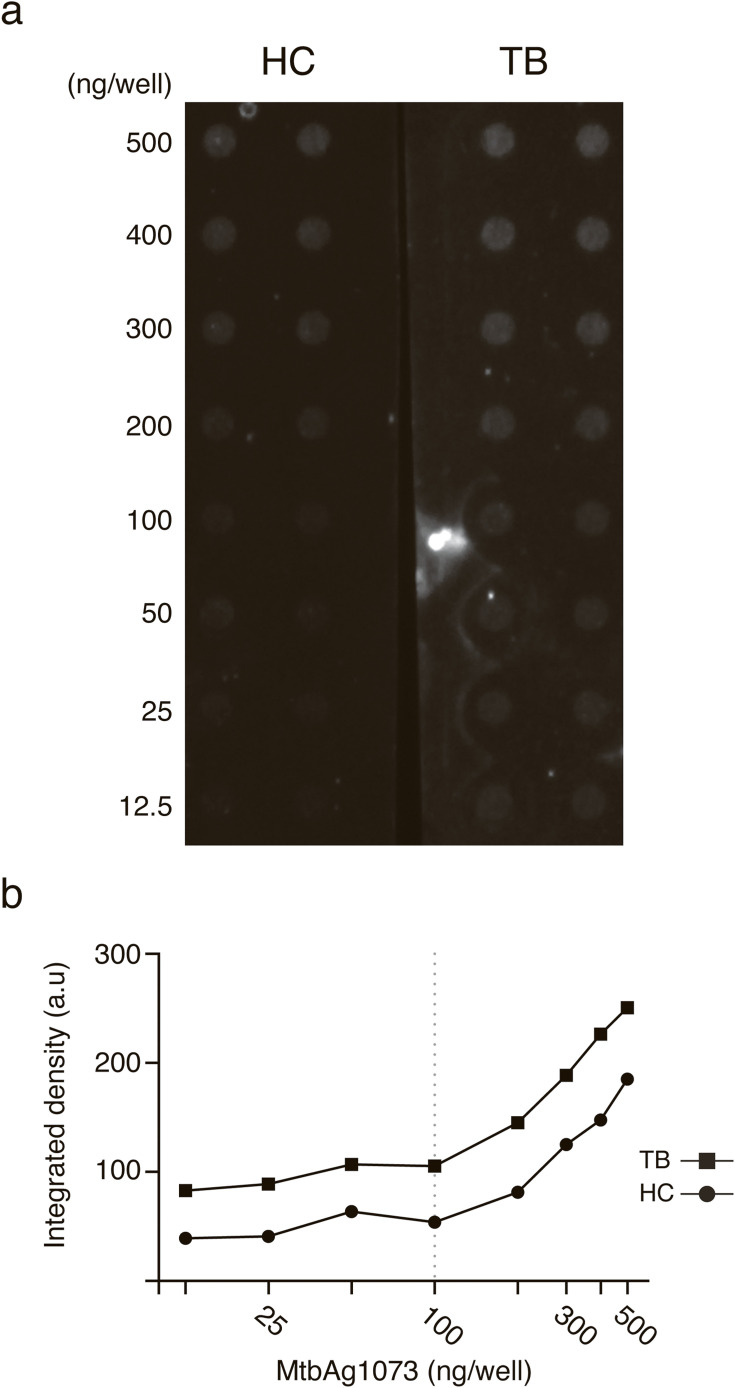
Optimization of antigen loading for the dot-blot detection of MtbAg1073. (a) Representative dot-blot images of recombinant MtbAg1073 spotted at the indicated concentrations (12.5–500 ng/well) and probed with pooled sera from healthy controls (HC) or tuberculosis (TB) patients. (b) Quantification of dot-blot signals shown in (a). Signal intensities were measured as integrated density using ImageJ. Circular regions of identical sizes were applied to each dot. Data are presented as raw signal intensities (arbitrary units, a.u.). The dotted vertical line indicates 100 ng/well, the concentration selected for subsequent experiments. Signals increased in a concentration-dependent manner within the linear detection range.

To select the appropriate antigen loading amount for the dot-blot assay, recombinant MtbAg1073 was spotted onto nitrocellulose membranes at concentrations ranging from 12.5 to 500 ng per well and probed with pooled sera from TB patients and HCs (Fig. 3A). Signal intensities increased in a concentration-dependent manner in TB sera, whereas only weak signals were observed with HC sera. Quantification of the dot-blot signals using ImageJ confirmed this result (Fig. 3B). Accordingly, 100 ng per well was selected for subsequent analyses because this amount provided sufficient signal intensity for TB sera while maintaining low background reactivity in HC and avoiding potential signal saturation at higher antigen concentrations.

### 6 Dot-blot analysis and diagnostic performance

#### 6.1 Serological evaluation by dot-blot and group-level discrimination

Initially, 45 TB and 42 HC serum samples were subjected to the dot-blot assay. After excluding samples based on the predefined criteria (Fig. S1), the final analysis was conducted on 40 TB and 40 HC samples.

**Fig. 4:**
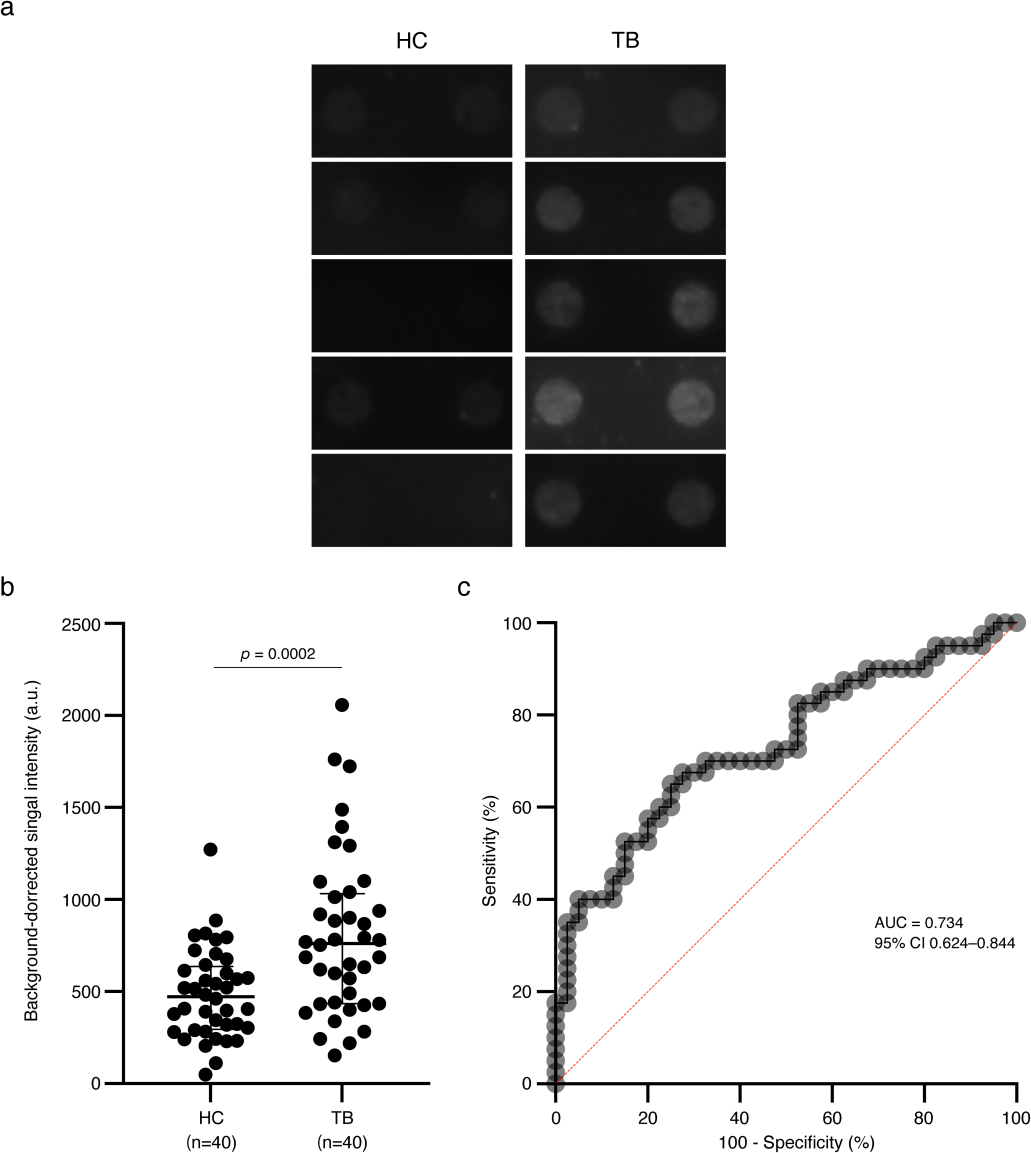
Serological reactivity of MtbAg1073 and diagnostic performance in tuberculosis patients. Samples with technical artifacts (TB: 3, HC: 1) were excluded prior to analysis, and three statistical outliers identified by the Grubbs’ test (TB: 2, HC: 1) were subsequently excluded. The sample selection process for dot-blot analysis is summarized in Fig. S1. (a) Representative dot-blot images obtained using individual sera from healthy controls (HC) and tuberculosis patients (TB). Recombinant MtbAg1073 (100 ng/well) was probed with sera diluted under identical conditions and detected by chemiluminescence. (b) Quantification of dot-blot signal intensities. Signal intensities were measured as background-corrected integrated density using ImageJ. Each dot represents an individual serum sample (HC, n = 40; TB, n = 40). Horizontal lines indicate median values, and error bars represent the interquartile range (IQR). The significance of differences was assessed using the Mann–Whitney U test. (c) A receiver operating characteristic (ROC) curve analysis evaluating the ability of anti-MtbAg1073 antibody responses to discriminate TB patients from healthy controls. The area under the curve (AUC) was 0.734 (95% CI: 0.624–0.844).

To evaluate the seroreactivity of MtbAg1073, a semi-quantitative dot-blot assay was performed using individual sera from microbiologically confirmed TB patients and HCs. Representative dot-blot results are shown in Fig. 4A.

The quantification of signal intensities revealed significantly higher antibody responses in the TB group than in the HC group (median: 761.2 vs. 471.9, the Mann-Whitney U test, *P* = 0.0002; Fig. 4B). Although signal intensities varied among TB patient sera, the overall distribution of values shifted toward higher reactivity in the TB group, indicating increased antibody responses against MtbAg1073 in active TB.

#### 6.2 ROC analysis

To further evaluate the diagnostic performance of MtbAg1073, a ROC curve analysis was performed (Fig. 4C). AUC was 0.734 (95% CI: 0.624–0.844, *P* = 0.0003), indicating a moderate ability to discriminate TB patients from HC.

Using the primary cut-off value selected at the point closest to the upper-left corner of the ROC curve, the assay achieved a sensitivity of 67.5% and specificity of 72.5%.

In addition, to explore a sensitivity-prioritized threshold, an alternative cut-off was defined as the point with the highest specificity among those maintaining a sensitivity of at least 70%. This approach resulted in a sensitivity of 70.0% and specificity of 67.5%. These results indicate that the diagnostic performance of MtbAg1073 varies depending on the selected threshold.

## Discussion

In the present study, we identified MtbAg1073, a conserved antigen belonging to the MTBC, through an immunoscreening strategy. By probing a cDNA expression library derived from *M. bovis* BCG Tokyo with sera from patients with active TB, we were able to identify antigens targeted by the host humoral immune response during infection. This strategy overcomes the limitations of candidate-based antigen discovery and enables the identification of antigens that are naturally recognized in human disease [17, 18]. Although MtbAg1073 is annotated solely as a conserved hypothetical protein in current genomic databases, with no prior report of antigenicity, the present study is the first to demonstrate its immunoreactivity with sera from active TB patients, providing experimental evidence that this protein is recognized by the human humoral immune response during infection.

Previous studies demonstrated that immunoscreening using genomic expression libraries identified *M. tuberculosis* antigens expressed *in vivo* during infection, including proteins containing repetitive amino acid motifs that exhibit strong serological immunoreactivity [19]. Similarly, studies employing recombinant protein expression enabled the identification of mycobacterial proteins capable of eliciting humoral immune responses in TB patients [17, 18]. Collectively, these studies indicate that immunoscreening is a useful approach for identifying diagnostically relevant antigens based on patterns of recognition by the human immune response.

One notable characteristic of MtbAg1073 is its high sequence conservation among representative MTBC strains, including *M. tuberculosis*, *M. bovis*, and *M. africanum*. This genetic stability is advantageous for the development of diagnostic antigens. Several mycobacterial antigens have been reported to exhibit sequence polymorphisms, which may lead to differences in antibody recognition and contribute to variability in the performance of serological diagnostics across populations and geographical regions [20–22]. Since signal peptides and transmembrane domains are not predicted for MtbAg1073, it is likely to be a cytoplasmic protein. Nevertheless, even intracellular mycobacterial proteins may be presented to the host immune system through bacterial lysis or antigen release during infection. These mechanisms have been reported for several immunoreactive mycobacterial antigens recognized by patient sera [19, 23].

Initial expression using conventional high-expression systems produced predominantly insoluble protein aggregates; therefore, a cold-shock expression system was adopted [24].

In contrast, the use of a cold-shock expression system enabled the production of soluble recombinant MtbAg1073. Obtaining the protein in its soluble form allows purification under native conditions and facilitates the preservation of both conformational and linear epitopes involved in antibody recognition [25]. In the present study, the successful production of soluble MtbAg1073 and its detectable immunoreactivity in TB patients support its potential as a candidate antigen for immunodiagnostic applications [26].

The serological evaluation revealed that antibodies against MtbAg1073 were associated with active TB. The Western blot analysis showed that sera from TB patients specifically recognized a band corresponding to the molecular weight of recombinant MtbAg1073, whereas no reactivity was observed with sera from HC. Furthermore, the dot-blot analysis enabled the semi-quantitative evaluation of antibody responses in a patient cohort from Lao PDR. The present results demonstrated that the TB patient group exhibited significantly higher signal intensities than the HC group (*P* = 0.0002).

The ROC analysis demonstrated moderate diagnostic performance (AUC = 0.734). In the initial evaluation of a novel antigen, this level of performance is consistent with the moderate performance typically reported for single antigen serological assays. For example, in a previous study by our research group, a dot-blot analysis using the MPB64 antigen achieved a sensitivity of 85.7% and specificity of 85.0% in serum samples [27]. Differences in diagnostic performance between antigens further underscore the variability inherent to single antigen serological assays.

In the present study, the primary cut-off, defined as the point closest to the upper-left corner of the ROC curve, yielded a sensitivity of 67.5% and specificity of 72.5%, indicating reasonably balanced diagnostic performance. In addition, a sensitivity-prioritized threshold, defined as the cut-off with the highest specificity among those maintaining a sensitivity of at least 70%, resulted in a sensitivity of 70.0% and specificity of 67.5%. These results demonstrate the consistent discriminatory ability of MtbAg1073 and suggest that threshold selection may be adjusted according to the intended clinical application.

Nevertheless, the present results also reflect the well-recognized limitations of single antigen serological approaches, particularly in the context of TB. Antibody responses in TB patients are known to markedly vary depending on a number of factors, such as the bacterial burden, stage of disease, and host immune status [5, 20], particularly in heterogeneous patient populations. The variability in signal intensities observed in this study is consistent with this heterogeneous nature of humoral immune responses in TB.

Strategies combining multiple antigens into diagnostic panels have been proposed to improve diagnostic performance by capturing complementary antibody responses [5, 20]. Therefore, MtbAg1073 may represent a potential component of future multi-antigen serological diagnostic panels.

This study has several limitations. First, the sample size was limited, and the case-control design comparing microbiologically confirmed TB patients with asymptomatic healthy controls may overestimate diagnostic performance compared with real-world clinical settings, in which such a test would typically be applied to patients with suspected TB or other respiratory conditions. Second, although the semi-quantitative dot-blot assay used in the present study is practical for initial antigen evaluation, it lacks the quantitative measurement capability of ELISA or chemiluminescent immunoassays. Third, the HC group was primarily defined based on clinical criteria, and latent TB infection was not microbiologically excluded. Since interferon-γ release assays or other immunological tests were not performed for control participants, the presence of subclinical or latent infection in some controls cannot be ruled out, which may have contributed to background antibody reactivity and affected specificity estimates. Fourth, since MtbAg1073 was identified from a BCG-derived library and its sequence is highly conserved across the MTBC, cross-reactivity in BCG-vaccinated individuals or those exposed to environmental mycobacteria cannot be excluded. In the present study, BCG vaccination status was not assessed in either group, and sera from BCG-vaccinated individuals without TB infection were not included. Therefore, whether serological testing using MtbAg1073 can discriminate active TB from BCG vaccination or environmental mycobacterial exposure remains to be determined, and future studies should include well-characterized BCG-vaccinated controls to address this question. Fifth, the age distribution and sex ratio differed between the TB and control groups; as antibody responses may vary according to host factors such as age and sex, confounding cannot be fully excluded. Finally, it remains uncertain whether the observed antibody responses are specific to active TB or may also be present in individuals with latent or prior TB infection. This is an inherent limitation of serological approaches, and future studies should include well-characterized latent TB cohorts to address this issue. Larger validation studies with more thoroughly characterized and age- and sex-matched control groups will be necessary to more accurately evaluate the diagnostic performance of this antigen.

Future studies need to focus on two directions. First, quantitative immunoassays, such as ELISA or chemiluminescent immunoassays, need to be developed in order to evaluate anti-MtbAg1073 antibody responses in larger patient cohorts. Second, the potential detection of antibodies against MtbAg1073 in urine warrants investigation. Urine represents an attractive clinical specimen for TB diagnostics, as it may be collected non-invasively without specialized medical procedures, and is particularly suitable for pediatric patients and resource-limited settings [28]. Tamada et al. [27] reported that antibody responses were detectable in both serum and urine using a dot-blot assay with a recombinant MPB64 antigen, and a strong correlation was observed between the two sample types. Therefore, antibody responses detectable in serum may also be present in urine [29], indicating the potential application of MtbAg1073 to urine-based diagnostic approaches.

## Conclusion

The present study identified a novel, conserved, and immunogenic antigen of the MTBC, MtbAg1073, through the unbiased immunoscreening of a cDNA expression library. The antigen was successfully expressed as a soluble recombinant protein. The present study is the first to demonstrate that MtbAg1073, a conserved MTBC protein previously annotated only as a hypothetical protein, elicits detectable antibody responses in active TB. These findings provide preliminary evidence of the immunoreactivity of MtbAg1073 in active TB and warrant further investigation into its potential utility in serological approaches, particularly in resource-limited settings where simple and accessible diagnostic tools are needed.

## Abbreviations

BCG: Bacillus Calmette–Guérin
LAM: Lipoarabinomannan
MTBC: *Mycobacterium tuberculosis* complex
TB: Tuberculosis
ROC: Receiver operating characteristic
AUC: Area under the curve
ELISA: Enzyme-linked immunosorbent assay
SDS-PAGE: Sodium dodecyl sulfate–polyacrylamide gel electrophoresis

## Supplementary information

**Additional file 1:** **Figure S1.** Flowchart of sample selection. The study cohort included 45 TB and 42 HC serum samples. One sample (marked as *a in Fig. S2) was excluded prior to group classification due to discordance between clinical presentation and GeneXpert MTB/RIF results. After removing technically invalid samples and statistical outliers, the final analysis included 40 TB and 40 HC samples.

**Additional file 2:** **Figure S2.** Raw dot-blot images of all analyzed serum samples. Dot-blot assays were performed in four independent runs (a–d). Pooled TB serum samples were included in each run as internal controls to monitor inter-assay variability. One sample (marked with an asterisk; *a) was excluded from quantitative analysis because of discordance between clinical presentation and GeneXpert MTB/RIF results. In addition, samples with excessively high background signals were excluded prior to quantification. Samples exhibiting irregular signal patterns were further evaluated based on the coefficient of variation (CV) between duplicate measurements. Samples with CV values exceeding 15% were visually inspected, and those with evident technical artifacts were excluded. Outlier detection was subsequently performed during statistical analysis, and the identified outliers were excluded from the final dataset. For selected healthy control samples (h30, h32, h34, and h35), duplicate measurements were performed across independent runs to assess reproducibility, and their mean values were used for quantitative analysis. Signal intensities were normalized across runs using pooled TB serum samples as internal controls. A scaling factor (calculated as 1000 divided by the mean signal intensity of the pooled TB control, averaged from duplicate measurements) was applied to each sample to enable comparison between runs.

## Declarations

### Availability of data and materials

All data generated or analyzed during this study are included in this published article.
