# Supplementary figures and images for: Identification and serological characterization of MtbAg1073, a conserved Mycobacterium tuberculosis complex antigen recognized in active tuberculosis
Source: Environ Health Prev Med. 2026 Jul 10;31:46. doi: 10.1265/ehpm.26-00071 (PMC13366182; doi:10.1265/ehpm.26-00071)

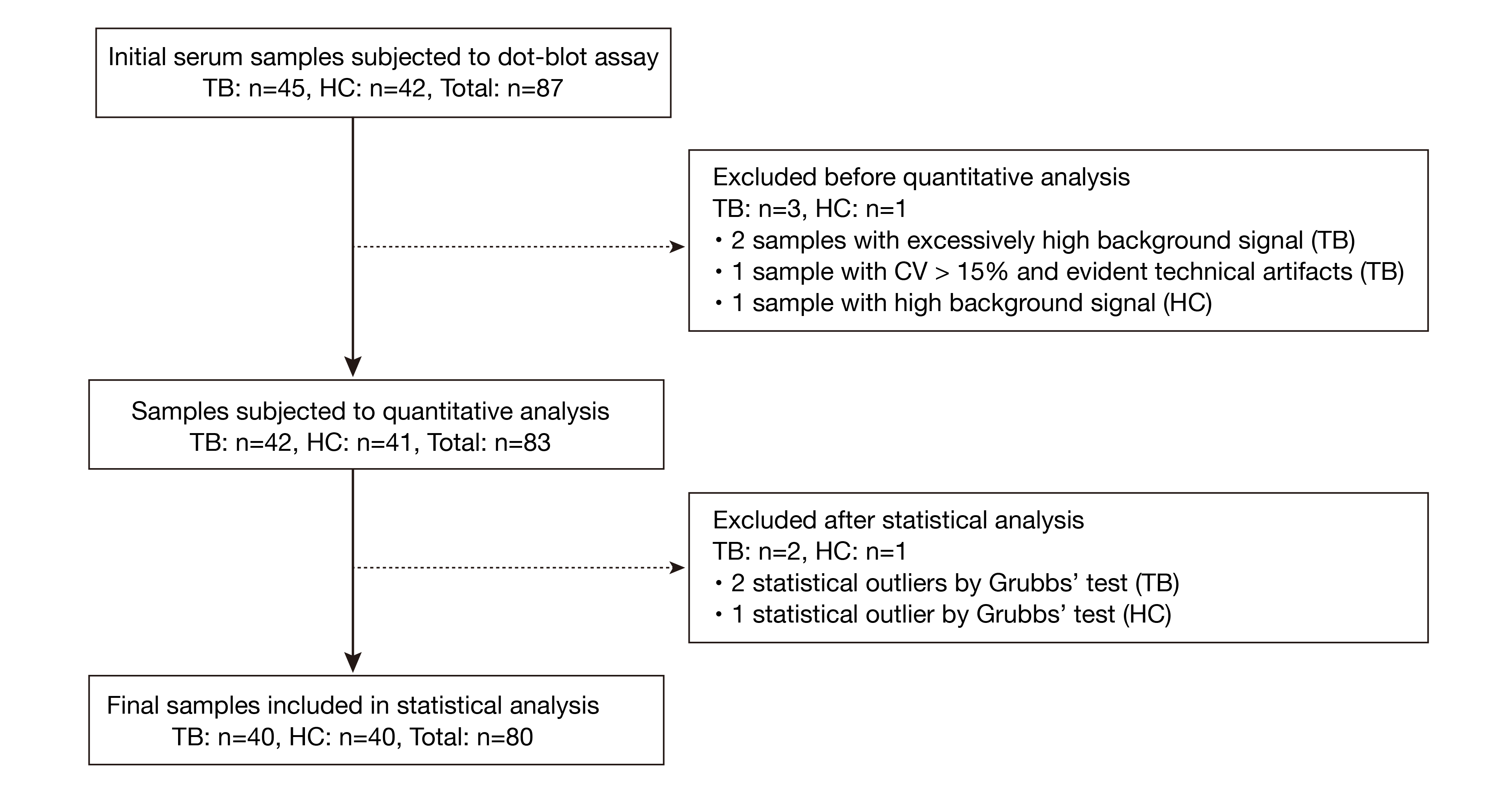

Supplement: Supplementary file 1 — Additional file 1: Figure S1. Flowchart of sample selection. The study cohort included 45 TB and 42 HC serum samples. One sample (marked as *a in Fig. S2) was excluded prior to group classification due to discordance between clinical presentation and GeneXpert MTB/RIF results. After removing technically invalid samples and statistical outliers, the final analysis included 40 TB and 40 HC samples. [file ehpm-31-046-s001.tif]

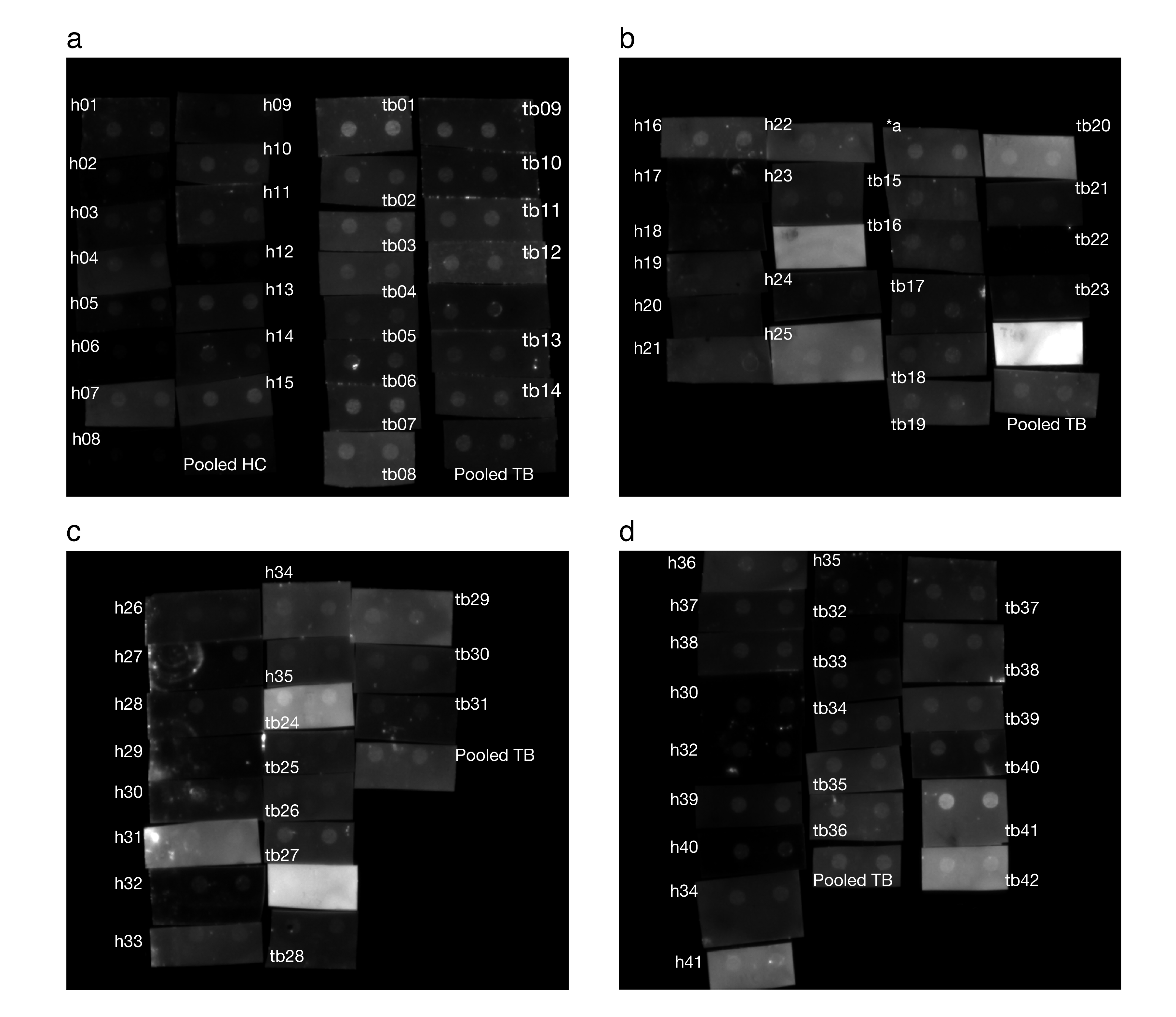

Supplement: Supplementary file 2 — Additional file 2: Figure S2. Raw dot-blot images of all analyzed serum samples. Dot-blot assays were performed in four independent runs (a–d). Pooled TB serum samples were included in each run as internal controls to monitor inter-assay variability. One sample (marked with an asterisk; *a) was excluded from quantitative analysis because of discordance between clinical presentation and GeneXpert MTB/RIF results. In addition, samples with excessively high background signals were excluded prior to quantification. Samples exhibiting irregular signal patterns were further evaluated based on the coefficient of variation (CV) between duplicate measurements. Samples with CV values exceeding 15% were visually inspected, and those with evident technical artifacts were excluded. Outlier detection was subsequently performed during statistical analysis, and the identified outliers were excluded from the final dataset. For selected healthy control samples (h30, h32, h34, and h35), duplicate measurements were performed across independent runs to assess reproducibility, and their mean values were used for quantitative analysis. Signal intensities were normalized across runs using pooled TB serum samples as internal controls. A scaling factor (calculated as 1000 divided by the mean signal intensity of the pooled TB control, averaged from duplicate measurements) was applied to each sample to enable comparison between runs. [file ehpm-31-046-s002.tif]
